# Genomic Epidemiology and Serology Associated with a SARS-CoV-2 R.1 Variant Outbreak in New Jersey

**DOI:** 10.1128/mbio.02141-22

**Published:** 2022-08-23

**Authors:** Barun Mathema, Liang Chen, Pengfei Wang, Marcus H. Cunningham, Jose R. Mediavilla, Kar Fai Chow, Yang Luo, Yanan Zhao, Kaelea Composto, Jerry Zuckerman, Michael C. Zody, Nancy Wilson, Annie Lee, Dayna M. Oschwald, Lihong Liu, Sho Iketani, Soren Germer, Samantha Fennessey, Maple Wang, Yael Kramer, Patricia Toole, Tom Maniatis, David D. Ho, David S. Perlin, Barry N. Kreiswirth

**Affiliations:** a Department of Epidemiology, Mailman School of Public Health, Columbia University grid.21729.3fgrid.239585.0 Irving Medical Center, New York, New York, USA; b Hackensack Meridian Health Center for Discovery and Innovation, Nutley, New Jersey, USA; c Hackensack Meridian School of Medicine, Nutley, New Jersey, USA; d Aaron Diamond AIDS Research Center, Columbia University grid.21729.3fgrid.239585.0 Vagelos College of Physicians and Surgeons, New York, New York, USA; e Hackensack Meridian Health Biorepository, Hackensack, New Jersey, USA; f Hackensack University Medical Center, Hackensack, New Jersey, USA; g Hackensack Meridian Health, Carrier Clinic, Belle Mead, New Jersey, USA; h New York Genome Center, New York, New York, USA; i Department of Microbiology and Immunology, Columbia University grid.21729.3fgrid.239585.0 Irving Medical Center, New York, New York, USA; j Division of Infectious Diseases, Department of Medicine, Columbia University grid.21729.3fgrid.239585.0 Vagelos College of Physicians and Surgeons, New York, New York, USA; Louis Stokes Veterans Affairs Medical Center

**Keywords:** SARS-CoV-2, variants of concern, spike protein, vaccine

## Abstract

Examining the neutralizing capacity of monoclonal antibodies (MAbs) used to treat COVID-19, as well as antibodies recovered from unvaccinated, previously vaccinated, and infected individuals, against severe acute respiratory syndrome coronavirus 2 (SARS-CoV-2) variants of concern (VOCs) remains critical to study. Here, we report on a SARS-CoV-2 nosocomial outbreak caused by the SARS-CoV-2 R.1 variant harboring the E484K mutation in a 281-bed psychiatric facility in New Jersey among unvaccinated inpatients and health care professionals (HCPs). A total of 81 inpatients and HCPs tested positive for SARS-Cov-2 by reverse transcription (RT)-PCR from 29 October 9 to 30 November 2020. The R.1 variant exhibits partial or complete resistance to two MAbs in clinical use, as well as 2 receptor binding domain MAbs and 4 N-terminal domain (NTD) MAbs. NTD MAbs against pseudovirus harboring single characteristic R.1 mutations highlight the role of S255F in loss of activity. Additionally, we note dampened neutralization capacity by plasma from individuals with previous SARS-CoV-2 infection or sera from vaccinated individuals. The relative resistance of the R.1 variant is likely lower than that of B.1.351 and closer to that of P.1 and B.1.526. The R.1 lineage has been reported in 47 states in the United States and 40 countries. Although high proportions exhibited symptoms (26% and 61% among patients and HCPs, respectively) and relative antibody resistance, we detected only 10 R.1 variants from over 2,900 samples (~0.34%) collected from January to October 2021. Among 3 vaccinated individuals previously infected with R.1, we observed robust neutralizing antibody responses against SARS-CoV-2 wild type and VOCs.

## INTRODUCTION

Severe acute respiratory syndrome coronavirus 2 (SARS-CoV-2) variants of concern (VOC), mutants that exhibit increased transmission, immune evasion, or both, have been reported in different global regions, and predominate lineages have been genotyped. Three VOCs, B.1.1.7 (Alpha) (1), B1.351 (Beta) (2), P.1 (Gamma) (3), and B.617.2 (Delta) (4), containing extensive mutations in the spike protein, emerged in the United Kingdom, South Africa, Brazil, and India, respectively, and have spread globally. Most recently, B.1.1.529 (Omicron) has dominated worldwide incident infections at an alarming pace (5, 6). The mapping of specific mutations in the spike protein has revealed strong evidence of convergent evolution, and in particular, the E484K change (or L452R and T478K in the case of Delta and S371L, N440K, G446S, and Q493R in the case of Omicron) (5, 7, 8), which enables evasion of monoclonal therapy and neutralizing antibodies, has been identified in discrete lineages in different geographic locations and associated with variant clones with increased transmission. The extent to which these VOC mutants diminish the efficacy of monoclonal therapy used to treat COVID-19 and the neutralizing capacity of antibodies recovered from unvaccinated, previously infected, and vaccinated individuals remains critical to study. In this study, we report on a SARS-CoV-2 nosocomial outbreak caused by the SARS-CoV-2 R.1 variant harboring the E484K mutation in a 281-bed psychiatric facility in New Jersey among unvaccinated inpatients and health care professionals (HCPs). We assessed R.1 and its S protein mutations with respect to antibody neutralization against 18 monoclonal antibodies (MAbs), including 5 with emergency use authorization (EUA), 20 convalescent-phase plasma samples, and 22 serum samples from vaccinated individuals.

## RESULTS

### Outbreak investigation.

**FIG 1:**
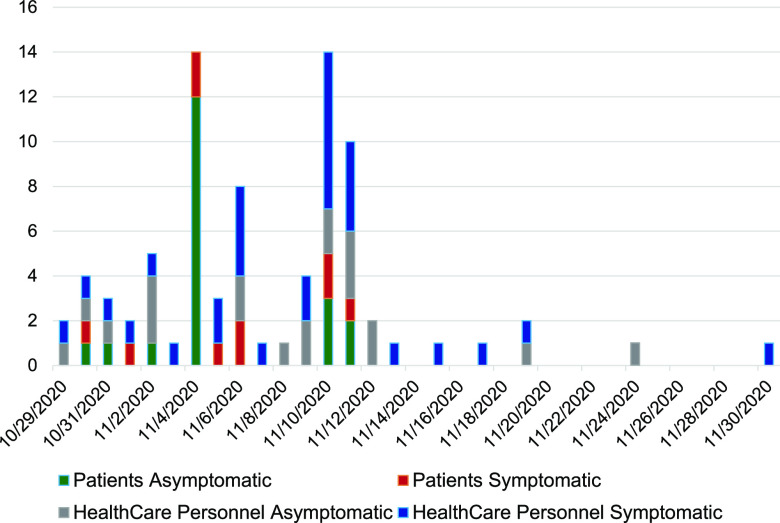
Epidemic curve of number of symptomatic and asymptomatic COVID-19 cases among health care professionals and patients detected over time at a clinic in New Jersey. Active screening for COVID-19 by RT-PCR occurred daily and included all inpatients and health care professionals regardless of symptoms. Dates on the *x* axis are given in month/day/year.

On 30 October 2020, the Hackensack Meridian Health (HMH) occupational health department in New Jersey was notified of 3 SARS-CoV-2-positive HCPs in the older adult unit (OAU), a 29-bed unit in an inpatient psychiatric 281-bed facility. All 29 OAU patients were subject to active screening for COVID-19 by reverse transcription (RT)-PCR. Active screening occurred daily and included all inpatients and HCPs. Rapid infection control, screening, and cohorting procedures were employed. Additionally, the OAU was closed for new admissions, inpatients were put on isolation precautions, visitation was changed to virtual only, congregate dining and group therapy were put on hold, and HCPs were assigned to only one unit (i.e., they did not attend multiple units). Over the next 4 weeks, 27/29 (93%) patients and 51/114 (45%) HCPs associated with the unit, including the electroconvulsive therapy and intensive care units, tested positive for SARS-CoV-2 by RT-PCR. In total, from 30 October 2020 until 30 November 2020, a total of 81 patients and HCPs tested positive (Fig. 1). Interestingly, asymptomatic infections occurred in 20/27 (74%) of the infected patients in the OAU, where the median age was 70 years, whereas only 20/51 (39%) were asymptomatic among the HCPs, where the median age was significantly lower, 38 years.

### WGS analyses indicate outbreak caused by SARS-CoV-2 R.1 lineage subvariant.

**FIG 2:**
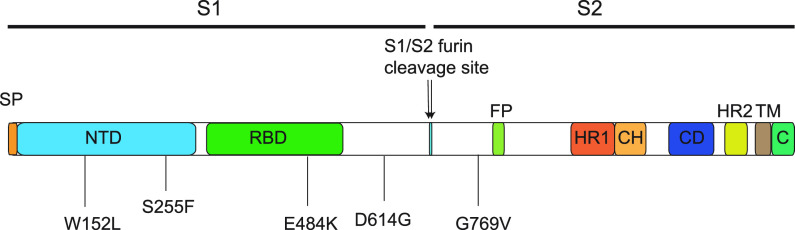
Schematic of SARS-CoV-2 spike protein primary structure of the R.1 outbreak strain. Different domains are shown by different colors. SP, signal peptide; NTD, N-terminal domain; RBD, receptor binding domain; S1, subdomain 1; S2, subdomain 2; FP, fusion peptide; HR1, heptad repeat 1; CH, central helix; CD, connector domain; HR2, heptad repeat 2; TM, transmembrane domain; C, cytoplasmic tail. The five S protein mutations and positions are illustrated below the S protein scheme.

We next assessed whether the epidemiology of the outbreak was supported by genomic data. Eleven retrospective viral samples from 11 HCPs in the OAU sampled between 6 November and 9 November were available for whole-genome sequencing (WGS). Analysis indicated closely related genomes (differing by 0 to 2 single nucleotide polymorphisms [SNPs]) consistent with transmission. Using established nomenclature, all 11 strains were classified as R.1 lineage, sublineage of B.1.1.316, and belonging to GR clade (GISAID) and 20B clade (Nextstrain). These strains had 15 or 16 amino acid substitutions and one nucleotide deletion (nt 28271A deletion) in comparison to the Wuhan-Hu-1 genome (GenBank accession no. MN908947.3). All strains had a set of common mutations in the spike protein: W152L, S255F, E484K, D614G, and G769V (Fig. 2). W152L and S255F reside in the antigenic supersite within the N-terminal domain (NTD) (9), which is a target for neutralizing antibodies, whereas E484K is situated at the receptor binding domain (RBD) interface with the cellular receptor ACE2. The G769V mutation is near the furin cleavage site (Fig. 2). In comparison to other global R.1 strains, the outbreak strains all have three unique nucleotide changes: T7685G (ORF1a:S2474A), A16569G (synonymous mutation), and C22326T (S:S255F). We suspected that most of the strains involved in this outbreak could be R.1. The rarity of R.1 and strains harboring E484K in New Jersey, the epidemic curve (Fig. 1), and finding 11 genomes from one facility are highly suggestive of extensive transmission. In addition, case findings initiated by infection control procedures (described above) and the high attack rate support the epidemiological linkage of COVID-19 cases suggestive of an outbreak.

### Global distribution of SARS-CoV-2 R.1 lineage.

**FIG 3:**
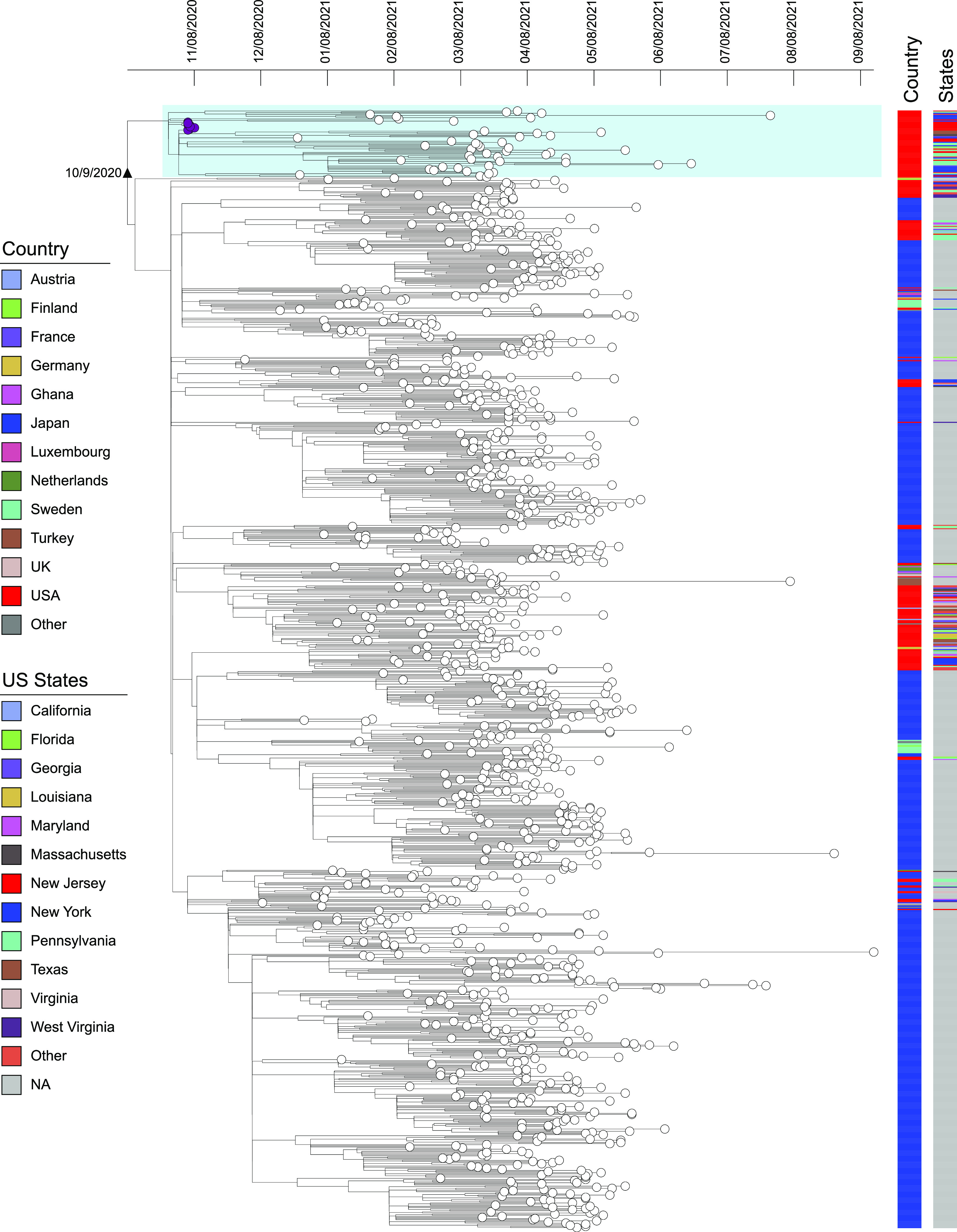
Bayesian maximum clade credibility trees of global SARS-Cov-2 R.1 lineage strains. Branch lengths are shown in years according to the scale bar at the top of each panel (dates given in month/day/year). Outbreak strains are colored in purple at the tips. The country and U.S. state information is illustrated by color bars on the right. The outbreak cluster with the S:S255F mutation is highlighted by light blue shading.

We assessed the global and U.S. distribution of the R.1 lineage uploaded to the GISAID database. As of 1 July 2022, there were 11,566 R.1 genomes reported from 40 countries, with Japan (*n* = 7,811, 67.5%) and the United States (*n* = 2,714, 23.5%) accounting for nearly 91% of all genomes. The R.1 lineage emerged in the middle of 2020, peaking around March 2021 in Japan, the United States, and other countries (see Fig. S1 in the supplemental material). Within the United States, 47 states have reported at least one case of R.1 infection. The outbreak strains (namely, outbreak cluster) were clustered with an additional ~600 strains, mostly from the United States (New York, New Jersey, Pennsylvania, Massachusetts, Connecticut, Florida, North Carolina, and Texas, etc.), as well as Canada (*n* = 47), Germany (*n* = 4), Japan (*n* = 2), Ghana (*n* = 1), and Finland (*n* = 1), in the GISAID database, and they all carry the S:S255F mutation. The time-resolved phylogeny of R.1 variants suggests that the outbreak cluster emerged and separated with other R.1 strains approximately 1 month prior to detection (Fig. 3). In addition, various R.1 sublineages, emerging almost at the same time as the outbreak cluster, were spreading globally in over 40 countries and in different U.S. states, suggesting that the ancestral strain acquired independent mutations within this short time span, followed by local transmission. Interestingly, the R.1 lineages gradually disappear from the middle of 2021, and the latest R.1 strain in the GISAID database was collected in November 2021.

10.1128/mbio.02141-22.1 FIG S1 Isolation times of R.1 strains in the GISAID database. Download FIG S1, PDF file, 0.4 MB. Copyright © 2022 Mathema et al. 2022 Mathema et al. https://creativecommons.org/licenses/by/4.0/ This content is distributed under the terms of the Creative Commons Attribution 4.0 International license.

### R.1 and pseudovirus antibody neutralization studies indicate evasion from most monoclonal antibodies.

**FIG 4:**
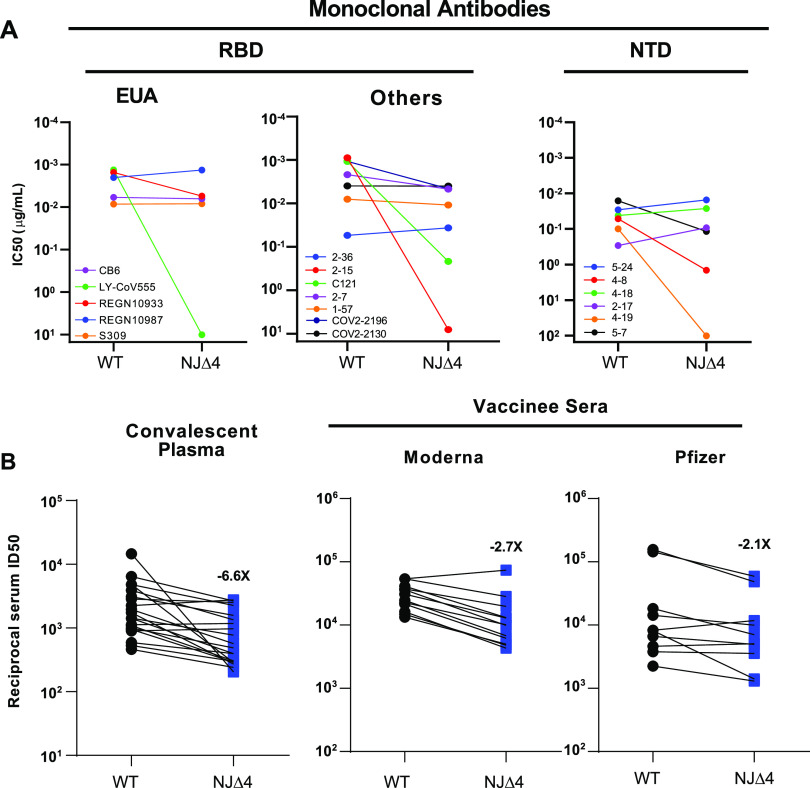
Neutralization of NJΔ4 (R.1) pseudoviruses by monoclonal antibodies, convalescent-phase plasma, and vaccinee sera. (A) Neutralization of NJΔ4 and WT (D614G) pseudoviruses by select MAbs. (B) Fold change in ID_50_ values for convalescent-phase plasma and vaccine sera against pseudoviruses of NJΔ4 relative to that of the WT. Data from 20 convalescent patients or 22 vaccinated individuals were averaged and are represented as the arithmetic mean ± standard error of the mean (SEM).

We next assessed the impact of the mutations in R.1 on antibody neutralization by using vesicular stomatitis virus (VSV)-based pseudovirus containing all four signature mutations (W152L, S255F, E484K, and G769V), termed NJΔ4. The mutant virus, in parallel with the wild-type (WT) (D614G) virus, was subjected to neutralization by 18 MAbs, including 5 with emergency use authorization (EUA), 20 convalescent-phase plasma samples, and 22 serum samples from vaccinated individuals. The specifics of these MAbs and clinical specimens were previously reported (10, 11). As shown in Fig. 4A (left panel) and Fig. S2A, the neutralizing activity of LY-CoV555 was completely abolished against R.1 and the activity of REGN10933 was also impaired, while the other three MAbs (REGN10987, CB6, and S309) with EUA retained their activities. We next tested the neutralizing activities of seven additional receptor binding domain (RBD) MAbs. The neutralizing activities of the two potent MAbs targeting the receptor binding motif, C121 and 2-15, were markedly or completely lost against R.1, while others retained their activities (Fig. 4A, middle panel, and Fig. S2A). These findings on R.1 mimic those observed for B.1.526 with the E484K mutation (12), indicating that the E484K mutation contributed to the lost activities of these RBD-directed MAbs. We then assessed the neutralizing activity of seven NTD MAbs against the R.1 pseudovirus (Fig. 4A, right panel, and Fig. S2B) (13). R.1 was profoundly resistant to neutralization by four NTD antibodies: 4-8, 4-19, 4A8, and 5-7, while the other three, 5-24, 4-18, and 2-17, remained active against R.1. To understand the specific mutations responsible for the observed pattern of neutralization, we then tested these NTD MAbs against a panel of pseudoviruses, each containing only a single NTD mutation found in R.1 (Fig. S2B). For the two NTD mutations, S255F contributed to the loss of activity of 4-8, 4-19, and 5-7, while W152L only partially accounted for the loss of activity of 4-19 and 5-7.

10.1128/mbio.02141-22.2 FIG S2 (A) Neutralization of WT and NJΔ4 (R.1) variant by anti-RBD MAbs. (B) Neutralization of WT and NJΔ4 single-mutation pseudoviruses by anti-NTD MAbs. Data represent the mean ± SEM of results of technical triplicates. Download FIG S2, PDF file, 1.5 MB. Copyright © 2022 Mathema et al. 2022 Mathema et al. https://creativecommons.org/licenses/by/4.0/ This content is distributed under the terms of the Creative Commons Attribution 4.0 International license.

### Neutralizing activity of sera from SARS-CoV-2 patients, vaccinated individuals, and R.1-infected and vaccinated individuals against R.1.

We also examined a panel of convalescent-phase plasma samples obtained from 20 SARS-CoV-2 patients (collected 1 month after documented infection) and vaccinee sera obtained from 12 individuals who had received the mRNA-1273 SARS-CoV-2 vaccine (collected on day 43; with two doses) and 10 individuals who had received the BNT162b2 COVID-19 vaccine (collected on day 28 or later; with two doses), as previously reported (10, 14, 15). Neutralizing activities of convalescent-phase plasma or vaccinee serum samples were lowered by 6.6-fold or 2.1- to 2.7-fold, respectively, against R.1 (Fig. 4B; Fig. S3 and S4). When the magnitude of resistance to R.1 of convalescent-phase plasma or vaccinee sera is compared with that to other variants of concern (10, 11), the relative resistance of R.1 is likely lower than that of B.1.351 and closer to that of P.1 and B.1.526.

10.1128/mbio.02141-22.3 FIG S3 Neutralization of WT and NJΔ4 (R.1) variant by convalescent-phase plasma. Data represent the mean ± SEM of results of technical triplicates. Download FIG S3, PDF file, 0.1 MB. Copyright © 2022 Mathema et al. 2022 Mathema et al. https://creativecommons.org/licenses/by/4.0/ This content is distributed under the terms of the Creative Commons Attribution 4.0 International license.

10.1128/mbio.02141-22.4 FIG S4 Neutralization of WT and NJΔ4 (R.1) variant by vaccine sera. Data represent the mean ± SEM of results of technical triplicates. Download FIG S4, PDF file, 0.2 MB. Copyright © 2022 Mathema et al. 2022 Mathema et al. https://creativecommons.org/licenses/by/4.0/ This content is distributed under the terms of the Creative Commons Attribution 4.0 International license.

**FIG 5:**
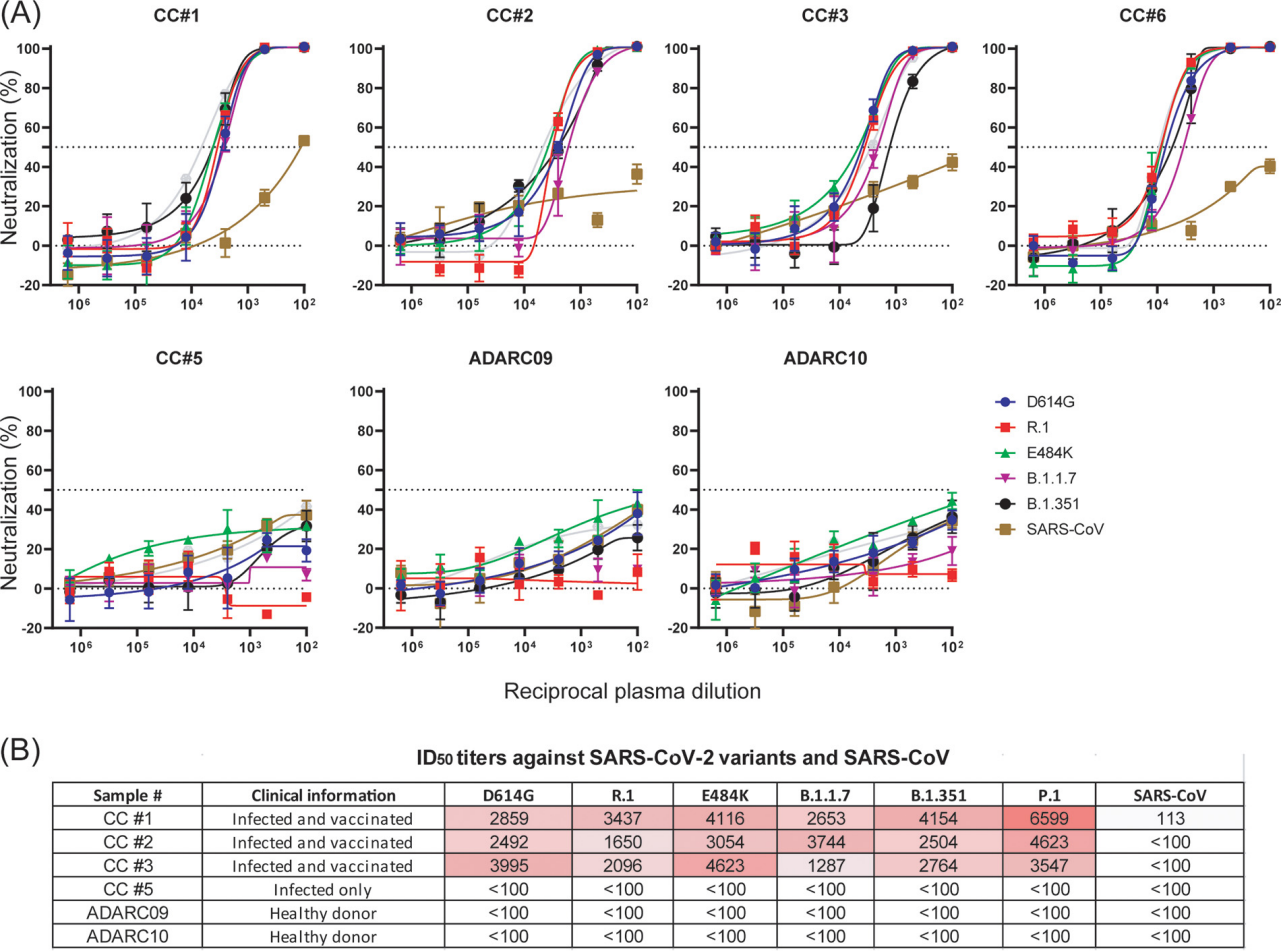
Pseudovirus neutralization by convalescent-phase sera. (A) Five patient serum samples were tested for neutralizing activity against VSV-G pseudotyped viruses with characteristic mutations: wild-type SARS-CoV-2 (D614G), SARS-CoV-2 R.1 (NJΔ4), SARS-CoV-2 variants B.1.1.7 and B.1.351, SARS-CoV-2 E484K, and SARS-CoV. CC#1, CC#2, CC#3, and CC#6 are patients who were infected with the R.1 variant and then received the Moderna vaccine (mRNA-1273). CC#5 is a patient only infected with the R.1 variant NJΔ4. ADARC09 and ADARC10 are serum samples from healthy individuals pre-SARS-CoV-2 pandemic that were used as negative controls. (B) ID_50_ values as reciprocal serum dilutions for each serum or antibody against different pseudoviruses are indicated. The limit of detection of this assay was 1:100 dilution.

We next assessed the neutralizing capacity of sera collected from four vaccinated HCPs previously infected with SARS-CoV-2 linked to the R.1 outbreak against major variants of concern, including B.1.1.7, P.1, and B.1.351 (Fig. 5). All four HCPs who were infected with the R.1 variant and had received two mRNA-1273 vaccinations displayed high titers against variants harboring E484K as well as the wild-type virus (Fig. 5A). Conversely, one HCP (CC#5) who was infected and symptomatic but not vaccinated failed to respond immunologically to either the E484K mutants or to the wild-type virus. Importantly, we found high titers against the wild type and major VOCs among those previously infected with the R.1 variant and then vaccinated. This latter observation is strong anecdotal evidence that the combination of infection and vaccination results in robust neutralizing antibody responses, consistent with recent studies (16, 17).

### Genomic screening for R.1 lineage reveals limited community spread.

To further investigate whether the R.1 variant outbreak was limited to the health care facility, we used a high-throughput molecular beacon assay to screen for E484K mutations in the RBD region (see Materials and Methods). From January 2021 to October 2021, nearly 2,900 positive COVID-19 swabs were stored and available for further testing. Overall, we detected 420 E484K mutants (420/2,879, 14.6%) (Fig. S5). The prevalence from January to May for E484K was 3.2% to 26.2% but decreased from June (18.1%) to August (0%). From August to October, all tested samples harbored L452R T478K double mutations, consistent with the genotype of the Delta variant.

10.1128/mbio.02141-22.5 FIG S5 Prevalence of E484K/Q, N501Y/T, L452R, and T478K mutants among SARS-Cov-2 samples from January 2021 to October 2021. Download FIG S5, PDF file, 0.1 MB. Copyright © 2022 Mathema et al. 2022 Mathema et al. https://creativecommons.org/licenses/by/4.0/ This content is distributed under the terms of the Creative Commons Attribution 4.0 International license.

To determine the phylogenetic background of E484K mutants, we conducted whole-genome sequencing on 112/401 E484K-positive but N501Y-negative mutants. Unlike with Japan, where R.1 has caused considerable community transmission (18), we detected only 10 R.1 variants, collected in February and March 2021, including 9 with the S255F mutation and belonging to the outbreak clusters from 6 different hospitals. Of the 112 E484K variants, 92% were of B.1.526 and related genetic backgrounds, consistent with the regional epidemiology in the Northeast during this time period (12). Since then, SARS-CoV-2 populations have been dominated by B.1.1.7 (with the N501Y mutation), which has since been displaced by Delta (B.617.2) and now Omicron (B.1.1.529).

## DISCUSSION

While health care institutions have experienced extensive SARS-CoV-2 transmission and high attack rates, they can also rapidly introduce effective infection control measures for containment. The R.1 outbreak in New Jersey, which occurred 2 months prior to vaccine availability, displayed an aggressive attack rate of 93% in residents and 34% in HCPs. Despite the extensive institutional spread, involving multiple medical units, patients, and HCPs, further transmission was limited. Prompt and responsive infection control measures (e.g., active case finding using SARS-CoV-2 PCR screening of all HCPs and patients, contact precautions, and restricted visitation, etc.) likely reduced further nosocomial transmission and dispersal into the community, evidenced by a sharp drop in incident infections at the health care facility and very few isolate (R.1 variant) cases detected in the community. In contrast, phylogeographic analysis of SARS-CoV-2 R.1 variants indicates wide geographic dispersal, including regional and coast-to-coast spread within the United States (Fig. 3). Of note, the acquisition of NTD mutation S255F, which is associated with loss of neutralization (Fig. S2B), is a key feature of the outbreak cluster and continental spread of SARS-CoV-2 R.1 variants in the United States and elsewhere.

More recently, the R.1 variant caused a large outbreak in a skilled nursing facility involving 46 residents and HCPs in Kentucky (19). This R.1 variant, harboring 4 of the 5 same spike protein mutations as the New Jersey variant (E484K, D614G, G769V, and W152L), spread among a population with 90% and 56% fully vaccinated residents and HCPs, respectively. Although estimation of vaccine effectiveness against infection is prone to study design effects, full vaccination afforded approximately 87% effectiveness for COVID-19-associated symptom prevention. Critically, high levels of COVID-19 symptom prevention were recorded despite R.1 variants harboring spike protein mutations (i.e., E484K and W152L). The New Jersey outbreak, in sharp contrast, registered symptoms in nearly 50% of SARS-CoV-2-positive, unvaccinated individuals.

These findings, along with serologic studies showing robust protection against VOCs among previously infected and vaccinated individuals, underscore the importance of vaccination for prevention of symptomatic COVID-19 disease. Most recently, the rapid expansion of Omicron is largely attributable to high transmissibility and antibody evasion potential among previously infected and vaccinated individuals (6, 20). However, previously infected and vaccinated groups appear to elicit neutralization capacity against Omicron consistent with our results (20).

## MATERIALS AND METHODS

### Clinical samples.

This observational study took place at a large hospital network in New Jersey. Nasopharyngeal swabs obtained as part of routine clinical care were tested by the clinical microbiology laboratory, and positive specimens were transferred to the Hackensack Meridian Health (HMH) BioRepository for storage. The study was reviewed and approved by the HMH Institutional Review Board (IRB) (Pro2020-0414). The IRB waived consent for the collection and sequencing of the viral samples as well as the abstraction of the epidemiologic metadata, as this study met the requirements for this exception. These include minimal risks to subjects, not adversely affecting the rights and welfare of the subjects, and that the research could not be carried out without the waiver. Under the IRB protocol (Pro2020-0414; approved 26 April 2020), health care workers were consenting to serology studies, including 10-mL whole-blood draws.

### Population sampling and screening of SARS-CoV-2.

SARS-CoV-2-positive swabs collected from seven HMH network hospitals were shipped to Center for Discovery and Innovation (Nutley, NJ) on a weekly basis and stored at −80°C. A total of ~2,879 swabs collected between January and October 2021 were subjected to a molecular beacon-based real-time asymmetric PCR and melting curve analysis to identify the SARS-CoV-2 E484K/Q, N501Y, L452R, and T478K mutations, by use of a previously described method (21).

### Whole-genome sequencing and phylogenetic analysis.

SARS-CoV-2-targeted assay libraries were prepared using the QIAseq FX DNA library (Qiagen) or AmpliSeq library plus and cDNA synthesis kits (Illumina) in accordance with the manufacturers’ recommendations. Briefly, 20 ng of RNA was reverse transcribed, followed by amplification of cDNA targets using the QIAseq or AmpliSeq SARS-CoV-2 research panel. Libraries were quantified using fluorescence-based assays, including PicoGreen (Life Technologies), a Qubit fluorometer (Invitrogen), and a fragment analyzer (Advanced Analytics). The final libraries were sequenced on a NovaSeq 6000 sequencer (v1 chemistry) with 2 × 150 bp.

Short-read data were filtered and processed prior to alignment. Read pairs that did not contain a single 19-bp seed k-mer in common with the SARS-CoV-2 genome reference (NC_045512.2) were discarded. Adapter sequences and low-quality (Phred quality score [*Q*] < 20) bases were trimmed from the remaining reads by using Cutadapt v2.10 (22). Processed reads were then mapped to the SARS-CoV-2 genome reference by using BWA-MEM v0.7.172 (23), and only read pairs with at least one alignment spanning a minimum of 42 bp in the reference and starting before position 29862 (to exclude polyadenine-only alignments) were kept. Genome sequences were determined by alignment pileup consensus calling with a minimum support of 20 reads and 95% breadth of coverage using Samtools v1.11 and bcftools v1.11 (23).

The resulting SARS-CoV-2 viral genome sequences were characterized by Nextclade CLI (v1) (https://clades.nextstrain.org/) to assign Nextstrain clades (24). SARS-CoV-2 lineage was determined using Pangolin v3.1.17 (https://github.com/cov-lineages/pangolin). The R.1 lineage genomes and metadata were downloaded from the GISAID website (*n* = 11,566, as of 1 July 2022). To conduct the phylogenetic analysis, only genomes marked as “complete” and “high coverage” in the GISAID metadata file and without ambiguous nucleotides (Ns) were included (*n* = 8,207). The genomes were aligned using Nextalign v1.10.0 (https://github.com/neherlab/nextalign), using the default setting, followed by removal of the 5′- and 3′-UTR regions. A maximum likelihood phylogenetic tree was constructed using IQ-TREE v2.1.2 (25) with automatic model selection and 1,000-bootstrap replicates. The tree was then reduced using Treemmer v0.3 (26) by keeping 50% relative tree length and at least one strain from each country and each U.S. state (*n* = 1,221). Further clock phylogenetic dating was conducted using treedater v0.5.0 (27). The resulting tree was annotated using ITOL v6 (28).

### Convalescent-phase serum samples and vaccinees.

Plasma samples were obtained from patients convalescing from documented SARS-CoV-2 infection approximately 1 month after recovery or later (10, 11). These cases were enrolled into an observational cohort study of convalescent patients followed at the Columbia University Irving Medical Center starting in the spring of 2020. The study protocol was approved by the Institutional Review Board (IRB), and all participants provided written informed consent. From their documented clinical profiles, 20 convalescent-phase plasma samples were selected for this study. Serum samples were obtained from 22 participants receiving either the mRNA-1273 SARS-CoV-2 vaccine at the NIH under an IRB-approved protocol or the Pfizer BNT162b2 COVID-19 vaccine as a part of the vaccine rollout among hospital employees (10, 11). These cases were then enrolled in an IRB-approved protocol to assess immunological responses to SARS-CoV-2.

### Monoclonal antibodies.

The monoclonal antibodies tested in this study were constructed and produced at Columbia University, except for REGN10933, REGN10987, COV2-2196, and COV2-2130, which were provided by Regeneron Pharmaceuticals, Inc., and CB6, which was provided by Baoshan Zhang and Peter D. Kwong (10).

### Construction and production of variant pseudoviruses.

The original pCMV3-SARS-CoV-2-spike plasmid was kindly provided by Peihui Wang of Shandong University in China. Plasmids encoding the D614G variant, all the single-mutation variants found in the R.1 variant, the 4-mutation combination variant (NJΔ4), and 8- and 9-mutation combination variants for B.1.1.7 and B.351, respectively, were generated by use of the QuikChange II XL site-directed mutagenesis kit (Agilent). Recombinant Indiana VSV (rVSV) expressing different SARS-CoV-2 spike variants was generated as previously described (10, 11). HEK293T cells were grown to 80% confluence before transfection with the spike gene using Lipofectamine 3000 (Invitrogen). Cells were cultured overnight at 37°C with 5% CO_2_, and VSV-G pseudotyped ΔG-luciferase (G*ΔG-luciferase; Kerafast) was used to infect the cells in Dulbecco’s modified Eagle’s medium (DMEM) at a multiplicity of infection (MOI) of 3 for 2 h before washing the cells with 1× Dulbecco’s phosphate-buffered saline (DPBS) three times. The next day, the transfection supernatant was harvested and clarified by centrifugation at 300 × *g* for 10 min. Each viral stock was then incubated with 20% I1 hybridoma (anti-VSV-G; ATCC CRL-2700) supernatant for 1 h at 37°C to neutralize contaminating VSV-G pseudotyped ΔG-luciferase virus before measuring titers and making aliquots to be stored at −80°C.

### Pseudovirus neutralization assays.

Neutralization assays were performed by incubating pseudoviruses with serial dilutions of MAbs or heat-inactivated plasma or serum and scored by the reduction in luciferase gene expression. In brief, Vero E6 cells were seeded in a 96-well plate at a concentration of 2 × 10^4^ cells per well. Pseudoviruses were incubated the next day with serial dilutions of the test samples in triplicate for 30 min at 37°C. The mixture was added to cultured cells and incubated for an additional 24 h. The luminescence was measured using the luciferase assay system (Promega). IC_50_ was defined as the dilution at which the relative light units were reduced by 50% compared to that of the virus control wells (virus plus cells) after subtraction of the background in the control groups with cells only (10, 11, 13). The IC_50_ values were calculated using nonlinear regression in GraphPad Prism.

### Data availability.

The SARS-CoV-2 genomes sequenced in this study were deposited in GISAID (https://www.gisaid.org). Sequences can be accessed by searching records from both the originating lab at Hackensack Medical Center and the submitting lab at the New York Genome Center.

## References

[1] Volz E, Mishra S, Chand M, Barrett JC, Johnson R, Geidelberg L, Hinsley WR, Laydon DJ, Dabrera G, O'Toole A, Amato R, Ragonnet-Cronin M, Harrison I, Jackson B, Ariani CV, Boyd O, Loman NJ, McCrone JT, Goncalves S, Jorgensen D, Myers R, Hill V, Jackson DK, Gaythorpe K, Groves N, Sillitoe J, Kwiatkowski DP, consortium C-GU, Flaxman S, Ratmann O, Bhatt S, Hopkins S, Gandy A, Rambaut A, Ferguson NM, COVID-19 Genomics UK (COG-UK) Consortium. 2021. Assessing transmissibility of SARS-CoV-2 lineage B.1.1.7 in England. Nature 593:266–269. doi:10.1038/s41586-021-03470-x. 33767447

[2] Tegally H, Wilkinson E, Giovanetti M, Iranzadeh A, Fonseca V, Giandhari J, Doolabh D, Pillay S, San EJ, Msomi N, Mlisana K, von Gottberg A, Walaza S, Allam M, Ismail A, Mohale T, Glass AJ, Engelbrecht S, Van Zyl G, Preiser W, Petruccione F, Sigal A, Hardie D, Marais G, Hsiao NY, Korsman S, Davies MA, Tyers L, Mudau I, York D, Maslo C, Goedhals D, Abrahams S, Laguda-Akingba O, Alisoltani-Dehkordi A, Godzik A, Wibmer CK, Sewell BT, Lourenco J, Alcantara LCJ, Kosakovsky Pond SL, Weaver S, Martin D, Lessells RJ, Bhiman JN, Williamson C, de Oliveira T. 2021. Detection of a SARS-CoV-2 variant of concern in South Africa. Nature 592:438–443. doi:10.1038/s41586-021-03402-9. 33690265

[3] Faria NR, Mellan TA, Whittaker C, Claro IM, Candido DDS, Mishra S, Crispim MAE, Sales FCS, Hawryluk I, McCrone JT, Hulswit RJG, Franco LAM, Ramundo MS, de Jesus JG, Andrade PS, Coletti TM, Ferreira GM, Silva CAM, Manuli ER, Pereira RHM, Peixoto PS, Kraemer MUG, Gaburo N, Jr, Camilo CDC, Hoeltgebaum H, Souza WM, Rocha EC, de Souza LM, de Pinho MC, Araujo LJT, Malta FSV, de Lima AB, Silva JDP, Zauli DAG, Ferreira ACS, Schnekenberg RP, Laydon DJ, Walker PGT, Schluter HM, Dos Santos ALP, Vidal MS, Del Caro VS, Filho RMF, Dos Santos HM, Aguiar RS, Proenca-Modena JL, Nelson B, Hay JA, Monod M, Miscouridou X, et al. 2021. Genomics and epidemiology of the P.1 SARS-CoV-2 lineage in Manaus, Brazil. Science 372:815–821. doi:10.1126/science.abh2644. 33853970 PMC8139423

[4] Liu J, Liu Y, Xia H, Zou J, Weaver SC, Swanson KA, Cai H, Cutler M, Cooper D, Muik A, Jansen KU, Sahin U, Xie X, Dormitzer PR, Shi PY. 2021. BNT162b2-elicited neutralization of B.1.617 and other SARS-CoV-2 variants. Nature 596:273–275. doi:10.1038/s41586-021-03693-y. 34111888

[5] Liu L, Iketani S, Guo Y, Chan JF, Wang M, Liu L, Luo Y, Chu H, Huang Y, Nair MS, Yu J, Chik KK, Yuen TT, Yoon C, To KK, Chen H, Yin MT, Sobieszczyk ME, Huang Y, Wang HH, Sheng Z, Yuen KY, Ho DD. 2022. Striking antibody evasion manifested by the omicron variant of SARS-CoV-2. Nature 602:676–681. doi:10.1038/d41586-021-03826-3. 35016198

[6] Yang W, Shaman J. 2021. SARS-CoV-2 transmission dynamics in South Africa and epidemiological characteristics of the Omicron variant. medRxiv.

[7] Planas D, Veyer D, Baidaliuk A, Staropoli I, Guivel-Benhassine F, Rajah MM, Planchais C, Porrot F, Robillard N, Puech J, Prot M, Gallais F, Gantner P, Velay A, Le Guen J, Kassis-Chikhani N, Edriss D, Belec L, Seve A, Courtellemont L, Pere H, Hocqueloux L, Fafi-Kremer S, Prazuck T, Mouquet H, Bruel T, Simon-Loriere E, Rey FA, Schwartz O. 2021. Reduced sensitivity of SARS-CoV-2 variant Delta to antibody neutralization. Nature 596:276–280. doi:10.1038/s41586-021-03777-9. 34237773

[8] Plante JA, Mitchell BM, Plante KS, Debbink K, Weaver SC, Menachery VD. 2021. The variant gambit: COVID-19's next move. Cell Host Microbe 29:508–515. doi:10.1016/j.chom.2021.02.020. 33789086 PMC7919536

[9] McCallum M, De Marco A, Lempp FA, Tortorici MA, Pinto D, Walls AC, Beltramello M, Chen A, Liu Z, Zatta F, Zepeda S, di Iulio J, Bowen JE, Montiel-Ruiz M, Zhou J, Rosen LE, Bianchi S, Guarino B, Fregni CS, Abdelnabi R, Foo SC, Rothlauf PW, Bloyet LM, Benigni F, Cameroni E, Neyts J, Riva A, Snell G, Telenti A, Whelan SPJ, Virgin HW, Corti D, Pizzuto MS, Veesler D. 2021. N-terminal domain antigenic mapping reveals a site of vulnerability for SARS-CoV-2. Cell 184:2332–2347.e2316. doi:10.1016/j.cell.2021.03.028. 33761326 PMC7962585

[10] Wang P, Nair MS, Liu L, Iketani S, Luo Y, Guo Y, Wang M, Yu J, Zhang B, Kwong PD, Graham BS, Mascola JR, Chang JY, Yin MT, Sobieszczyk M, Kyratsous CA, Shapiro L, Sheng Z, Huang Y, Ho DD. 2021. Antibody resistance of SARS-CoV-2 variants B.1.351 and B.1.1.7. Nature 593:130–135. doi:10.1038/s41586-021-03398-2. 33684923

[11] Wang P, Casner RG, Nair MS, Wang M, Yu J, Cerutti G, Liu L, Kwong PD, Huang Y, Shapiro L, Ho DD. 2021. Increased resistance of SARS-CoV-2 variant P.1 to antibody neutralization. Cell Host Microbe 29:747–751.e744. doi:10.1016/j.chom.2021.04.007. 33887205 PMC8053237

[12] Annavajhala MK, Mohri H, Wang P, Nair M, Zucker JE, Sheng Z, Gomez-Simmonds A, Kelley AL, Tagliavia M, Huang Y, Bedford T, Ho DD, Uhlemann AC. 2021. Emergence and expansion of SARS-CoV-2 B.1.526 after identification in New York. Nature 597:703–708. doi:10.1038/s41586-021-03908-2. 34428777 PMC8481122

[13] Liu L, Wang P, Nair MS, Yu J, Rapp M, Wang Q, Luo Y, Chan JF, Sahi V, Figueroa A, Guo XV, Cerutti G, Bimela J, Gorman J, Zhou T, Chen Z, Yuen KY, Kwong PD, Sodroski JG, Yin MT, Sheng Z, Huang Y, Shapiro L, Ho DD. 2020. Potent neutralizing antibodies against multiple epitopes on SARS-CoV-2 spike. Nature 584:450–456. doi:10.1038/s41586-020-2571-7. 32698192

[14] Polack FP, Thomas SJ, Kitchin N, Absalon J, Gurtman A, Lockhart S, Perez JL, Pérez Marc G, Moreira ED, Zerbini C, Bailey R, Swanson KA, Roychoudhury S, Koury K, Li P, Kalina WV, Cooper D, Frenck RW, Hammitt LL, Türeci Ö, Nell H, Schaefer A, Ünal S, Tresnan DB, Mather S, Dormitzer PR, Şahin U, Jansen KU, Gruber WC, C4591001 Clinical Trial Group. 2020. Safety and efficacy of the BNT162b2 mRNA Covid-19 vaccine. N Engl J Med 383:2603–2615. doi:10.1056/NEJMoa2034577. 33301246 PMC7745181

[15] Anderson EJ, Rouphael NG, Widge AT, Jackson LA, Roberts PC, Makhene M, Chappell JD, Denison MR, Stevens LJ, Pruijssers AJ, McDermott AB, Flach B, Lin BC, Doria-Rose NA, O'Dell S, Schmidt SD, Corbett KS, Swanson PA, 2nd, Padilla M, Neuzil KM, Bennett H, Leav B, Makowski M, Albert J, Cross K, Edara VV, Floyd K, Suthar MS, Martinez DR, Baric R, Buchanan W, Luke CJ, Phadke VK, Rostad CA, Ledgerwood JE, Graham BS, Beigel JH, mRNA-1273 Study Group. 2020. Safety and immunogenicity of SARS-CoV-2 mRNA-1273 vaccine in older adults. N Engl J Med 383:2427–2438. doi:10.1056/NEJMoa2028436. 32991794 PMC7556339

[16] Cho A, Muecksch F, Schaefer-Babajew D, Wang Z, Finkin S, Gaebler C, Ramos V, Cipolla M, Mendoza P, Agudelo M, Bednarski E, DaSilva J, Shimeliovich I, Dizon J, Daga M, Millard K, Turroja M, Schmidt F, Zhang F, Tanfous TB, Jankovic M, Oliveria TY, Gazumyan A, Caskey M, Bieniasz PD, Hatziioannou T, Nussenzweig MC. 2021. Anti-SARS-CoV-2 receptor binding domain antibody evolution after mRNA vaccination. Nature 600:517–522. doi:10.1038/s41586-021-04060-7. 34619745 PMC8674133

[17] Sokal A, Barba-Spaeth G, Fernandez I, Broketa M, Azzaoui I, de La Selle A, Vandenberghe A, Fourati S, Roeser A, Meola A, Bouvier-Alias M, Crickx E, Languille L, Michel M, Godeau B, Gallien S, Melica G, Nguyen Y, Zarrouk V, Canoui-Poitrine F, Pirenne F, Megret J, Pawlotsky JM, Fillatreau S, Bruhns P, Rey FA, Weill JC, Reynaud CA, Chappert P, Mahevas M. 2021. mRNA vaccination of naive and COVID-19-recovered individuals elicits potent memory B cells that recognize SARS-CoV-2 variants. Immunity 54:2893–2907.e5. doi:10.1016/j.immuni.2021.09.011. 34614412 PMC8452492

[18] Sekizuka T, Itokawa K, Hashino M, Okubo K, Ohnishi A, Goto K, Tsukagoshi H, Ehara H, Nomoto R, Ohnishi M, Kuroda M, Virus Diagnosis Group (NIID Toyama), COVID-19 Genomic Surveillance Network in Japan (COG-JP). 2021. A discernable increase in the severe acute respiratory syndrome coronavirus 2 R.1 lineage carrying an E484K spike protein mutation in Japan. Infect Genet Evol 94:105013. doi:10.1016/j.meegid.2021.105013. 34352360 PMC8327703

[19] Cavanaugh AM, Fortier S, Lewis P, Arora V, Johnson M, George K, Tobias J, Lunn S, Miller T, Thoroughman D, Spicer KB. 2021. COVID-19 outbreak associated with a SARS-CoV-2 R.1 lineage variant in a skilled nursing facility after vaccination program—Kentucky, March 2021. MMWR Morb Mortal Wkly Rep 70:639–643. doi:10.15585/mmwr.mm7017e2. 33914720 PMC8084128

[20] Cele S, Jackson L, Khoury DS, Khan K, Moyo-Gwete T, Tegally H, San JE, Cromer D, Scheepers C, Amoako DG, Karim F, Bernstein M, Lustig G, Archary D, Smith M, Ganga Y, Jule Z, Reedoy K, Hwa S-H, Giandhari J, Blackburn JM, Gosnell BI, Abdool Karim SS, Hanekom W, Davies M-A, Hsiao M, Martin D, Mlisana K, Wibmer CK, Williamson C, York D, Harrichandparsad R, Herbst K, Jeena P, Khoza T, Kløverpris H, Leslie A, Madansein R, Magula N, Manickchund N, Marakalala M, Mazibuko M, Moshabela M, Mthabela N, Naidoo K, Ndhlovu Z, Ndung’u T, Ngcobo N, Nyamande K, Patel V, NGS-SA, et al. 2022. Omicron extensively but incompletely escapes Pfizer BNT162b2 neutralization. Nature 602:654–656. doi:10.1038/d41586-021-03824-5. 35016196 PMC8866126

[21] Zhao Y, Lee A, Composto K, Cunningham MH, Mediavilla JR, Fennessey S, Corvelo A, Chow KF, Zody M, Chen L, Kreiswirth BN, Perlin DS. 2021. A novel diagnostic test to screen SARS-CoV-2 variants containing E484K and N501Y mutations. Emerg Microbes Infect 10:994–997. doi:10.1080/22221751.2021.1929504. 33977858 PMC8168736

[22] Kechin A, Boyarskikh U, Kel A, Filipenko M. 2017. cutPrimers: a new tool for accurate cutting of primers from reads of targeted next generation sequencing. J Comput Biol 24:1138–1143. doi:10.1089/cmb.2017.0096. 28715235

[23] Li H, Durbin R. 2009. Fast and accurate short read alignment with Burrows-Wheeler transform. Bioinformatics 25:1754–1760. doi:10.1093/bioinformatics/btp324. 19451168 PMC2705234

[24] Hadfield J, Megill C, Bell SM, Huddleston J, Potter B, Callender C, Sagulenko P, Bedford T, Neher RA. 2018. Nextstrain: real-time tracking of pathogen evolution. Bioinformatics 34:4121–4123. doi:10.1093/bioinformatics/bty407. 29790939 PMC6247931

[25] Nguyen LT, Schmidt HA, von Haeseler A, Minh BQ. 2015. IQ-TREE: a fast and effective stochastic algorithm for estimating maximum-likelihood phylogenies. Mol Biol Evol 32:268–274. doi:10.1093/molbev/msu300. 25371430 PMC4271533

[26] Menardo F, Loiseau C, Brites D, Coscolla M, Gygli SM, Rutaihwa LK, Trauner A, Beisel C, Borrell S, Gagneux S. 2018. Treemmer: a tool to reduce large phylogenetic datasets with minimal loss of diversity. BMC Bioinformatics 19:164. doi:10.1186/s12859-018-2164-8. 29716518 PMC5930393

[27] Volz EM, Frost SDW. 2017. Scalable relaxed clock phylogenetic dating. Virus Evol 3:vex025. doi:10.1093/ve/vex025.

[28] Letunic I, Bork P. 2019. Interactive Tree Of Life (iTOL) v4: recent updates and new developments. Nucleic Acids Res 47:W256–W259. doi:10.1093/nar/gkz239. 30931475 PMC6602468

