# Supplementary figures and images for: Genomic Epidemiology and Serology Associated with a SARS-CoV-2 R.1 Variant Outbreak in New Jersey
Source: mBio. 2022 Aug 23;13(5):e02141-22. doi: 10.1128/mbio.02141-22 (PMC9600516; doi:10.1128/mbio.02141-22)

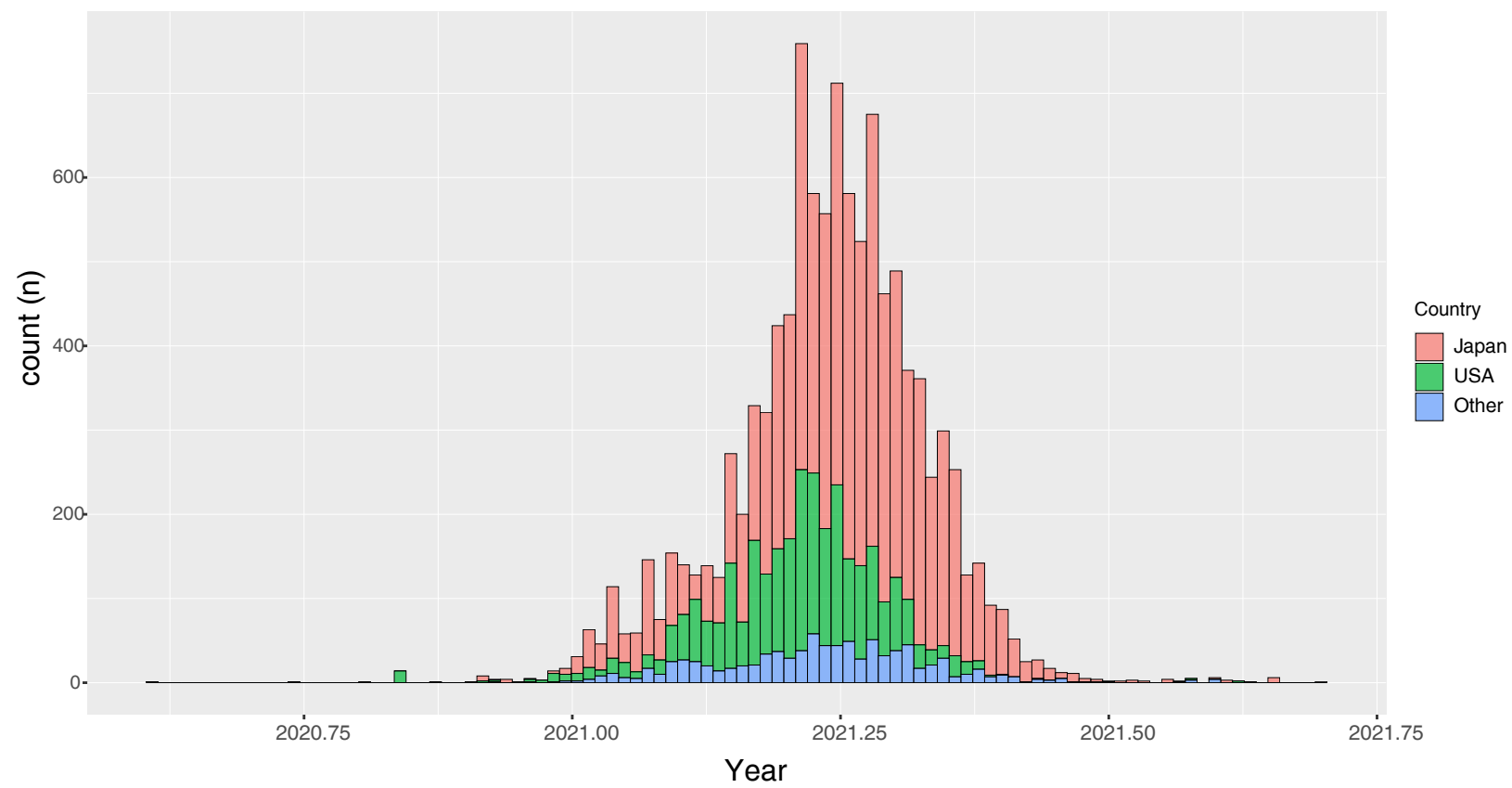

Supplement: FIG S1 [file mbio.02141-22-s0001.pdf]

(A)

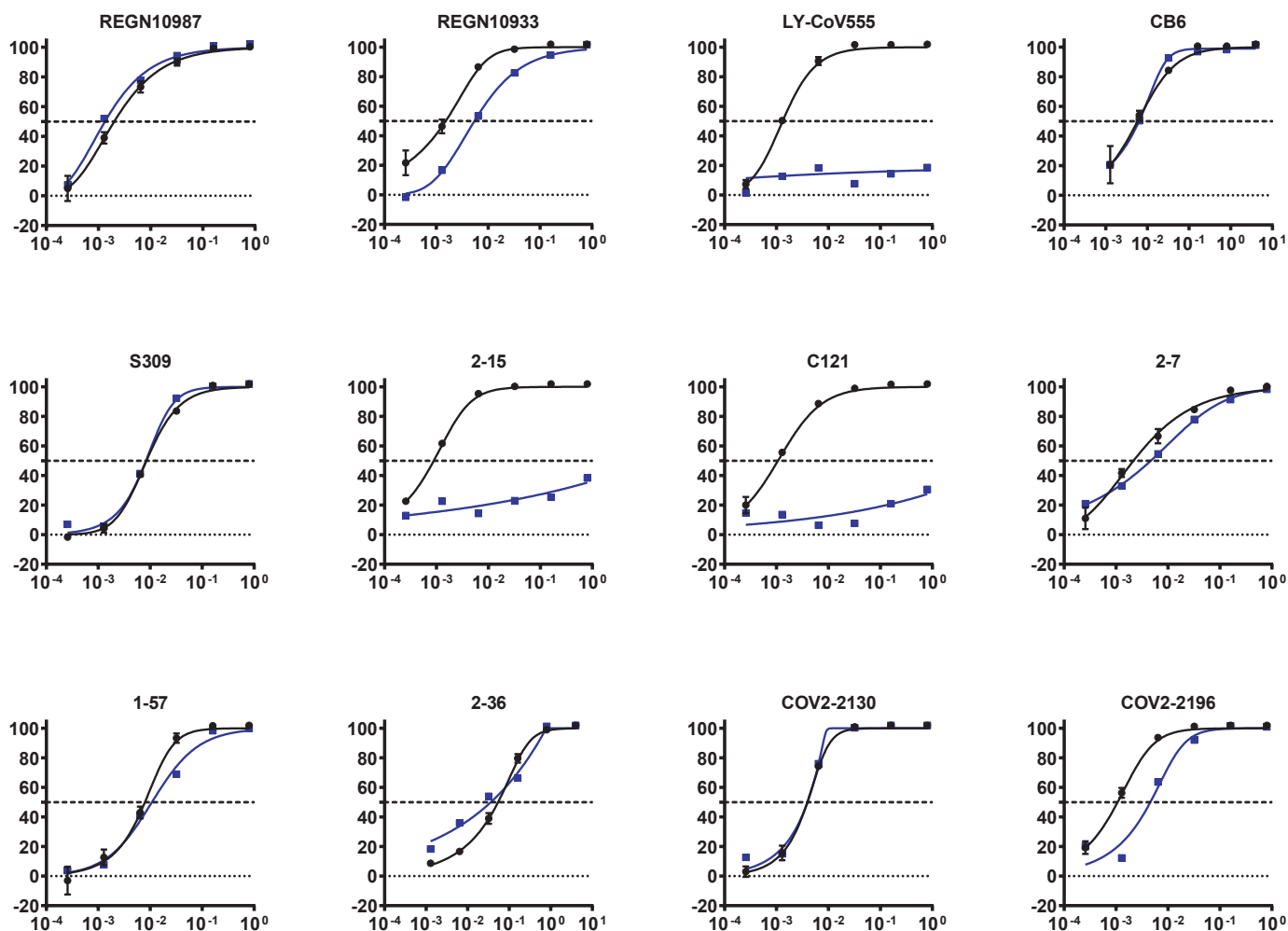

(B)

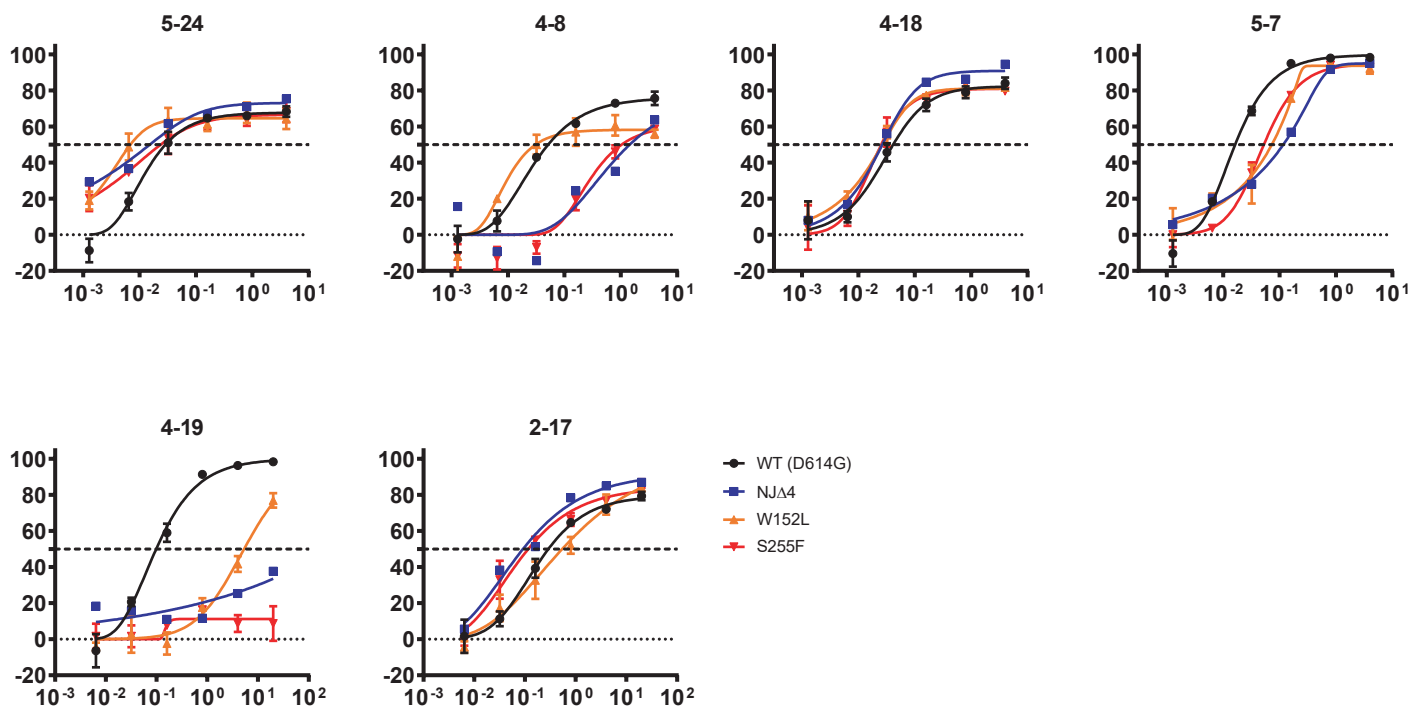

Supplement: FIG S2 [file mbio.02141-22-s0002.pdf]

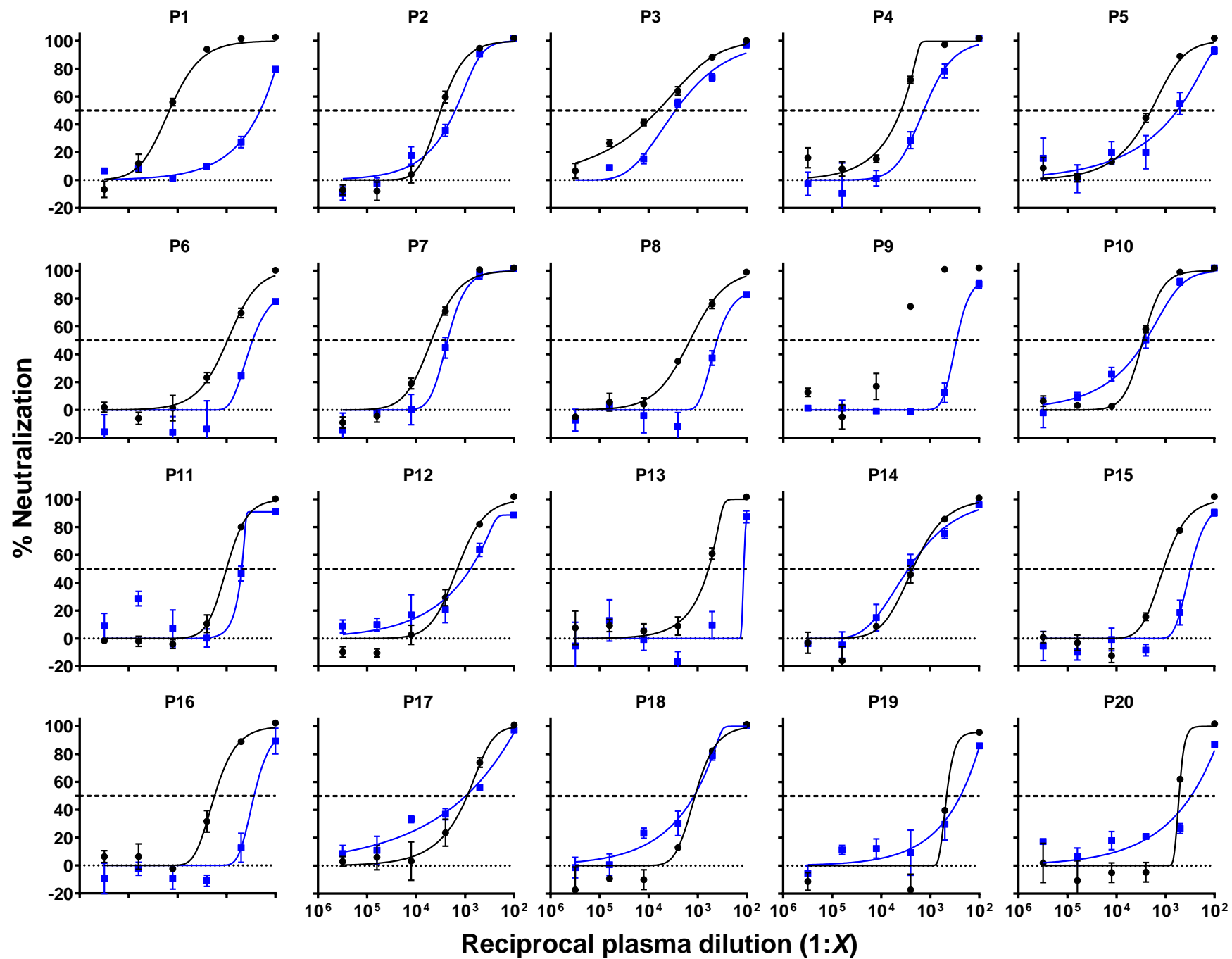

Supplement: FIG S3 [file mbio.02141-22-s0003.pdf]

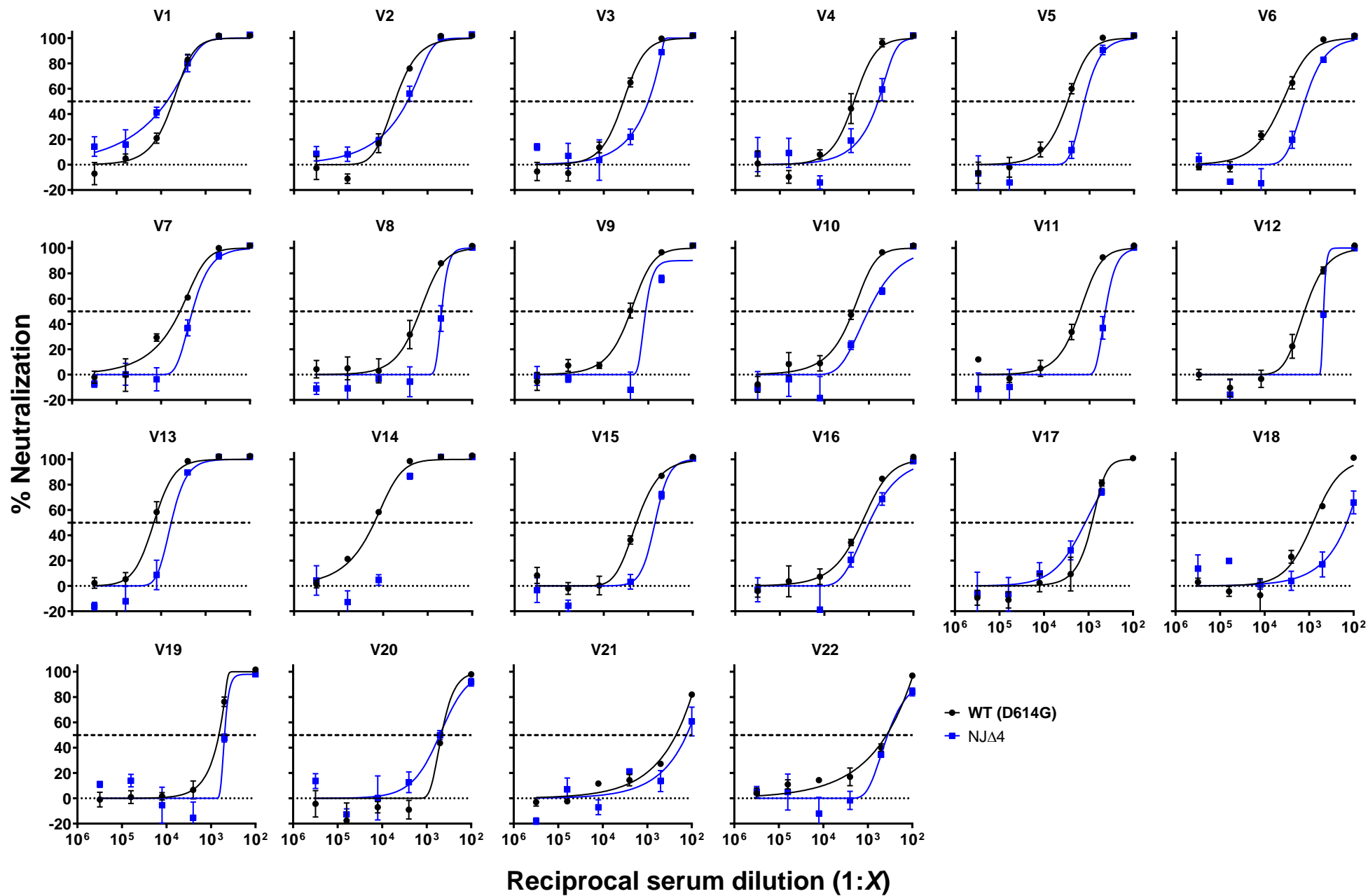

Supplement: FIG S4 [file mbio.02141-22-s0004.pdf]

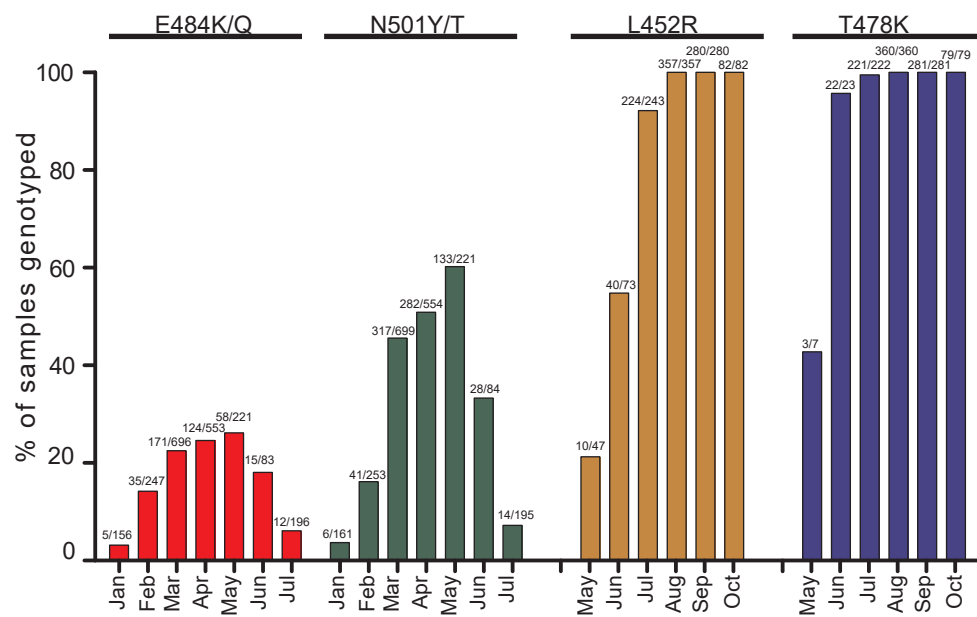

Supplement: FIG S5 [file mbio.02141-22-s0005.pdf]
